# In Vitro Evaluation of Sodium Hypochlorite, Chlorhexidine, Propolis, and Calcium Hydroxide Effect on Lipoteichoic-Acid-Induced Proinflammatory Cytokines Production

**DOI:** 10.3390/dj12090286

**Published:** 2024-09-05

**Authors:** Luciane Dias de Oliveira, Lara Steffany de Carvalho, Ana Claudia Carvalho Xavier, Felipe Eduardo de Oliveira, Mariella Vieira Pereira Leão, Mariana Gadelho Gimenez Diamantino, Rayana Duarte Khoury, Marcia Carneiro Valera, Cláudio Antonio Talge Carvalho, Amjad Abu Hasna

**Affiliations:** 1 Department of Bioscience and Oral Diagnosis, Institute of Science and Technology, Campus of São José dos Campos, São Paulo State University, São Paulo 12245-000, Brazil; luciane.oliveira@unesp.br (L.D.d.O.); larasteffany2011@gmail.com (L.S.d.C.); mariellaleao@yahoo.com.br (M.V.P.L.); 2 Department of Restorative Dentistry, Endodontics Division, Institute of Science and Technology, Campus of São José dos Campos, São Paulo State University, São Paulo 12245-000, Brazil; mariana.gimenez@unesp.br (M.G.G.D.); rayana.khoury@gmail.com (R.D.K.); marcia.valera@unesp.br (M.C.V.); claudio.talge@unesp.br (C.A.T.C.); 3 School of Dentistry, Universidad Espíritu Santo, Samborondón 092301, Ecuador

**Keywords:** calcium hydroxide, chlorhexidine, cytokines, *Enterococcus faecalis*, lipoteichoic acid, propolis, sodium hypochlorite

## Abstract

This study aimed to evaluate the effects of sodium hypochlorite (NaOCl), chlorhexidine (CHX), and the glycolic extract of propolis (GEP) as endodontic irrigants and of calcium hydroxide [Ca(OH)_2_], CHX, or Ca(OH)_2_ + CHX as intracanal medications on the capacity of the lipoteichoic acid (LTA) of *Enterococcus faecalis* in macrophages’ proinflammatory cytokines production. Freshly extracted 108 human single-rooted teeth were used in this study. The LTA of *E. faecalis* was standardized in double-distilled pyrogen-free water (250 µg/mL) and inoculated into the specimens subdivided into nine subgroups (n = 12). Cultures of murine macrophages (RAW 264.7) were treated with 30 µL of each sample collected from root canals and incubated (37 °C, 5% CO_2_) for 24 h. Lastly, anti-TNF-α, anti-IL-6, anti-IP-10, anti-MIP-1α, anti-G-CSF, and anti-IL-1β DuoSet kits were used to perform an ELISA assay. Data were analyzed using one-way ANOVA and Tukey test (*p* ≥ 0.05). It was found that 1% NaOCl was the most effective irrigant in reducing the capacity of LTA in cytokines production, followed by 12% GEP and 2% CHX, respectively. Ca(OH)_2_ + CHX presented the best results when associated with NaOCl or GEP. Thus, NaOCl or GEP associated with Ca(OH)_2_ + CHX were effective in reducing the capacity of LTA in different macrophages pro-inflammatory cytokines production.

## 1. Introduction

*Enterococcus faecalis* is a Gram-positive bacterium. It is involved in primary and secondary endodontic infections [1]. It is considered to be the main reason for endodontic failure [2]. Its endotoxin, the lipoteichoic acid (LTA), is found in the outer leaflet of the plasma membrane of the bacterial cell [3]. It has a role in TLR2 (toll-like receptor 2) receptor activation [4]. LTA has been described as the main antigen involved in the stimulation of tumor necrosis factor-alpha (TNF-α) production by macrophages, alongside its role in inducing the production of several pro-inflammatory cytokines in macrophages [5,6].

Sodium hypochlorite (NaOCl) is a widely used endodontic irrigant. It has antimicrobial action against diverse microorganisms, it detoxifies endotoxins and disactivates matrix metalloproteinases (MMP) [7], and it dissolves organic pulp tissues of the root canal [8]. It primarily works through the formation of hypochlorous acid (HOCl) when dissolved in water, which penetrates the cell walls and disrupts cellular components and can neutralize bacterial endotoxins, reducing their inflammatory effects [9]. Alongside chlorhexidine (CHX), which is another antimicrobial agent used in endodontics as an irrigant with saline solution or as an intracanal dressing, it has a wide antimicrobial action and antiendotoxin activity [10,11]. It has cationic properties, allowing it to bind to negatively charged bacterial cell walls. This disrupts cell membrane integrity, leading to the leakage of cellular contents and cell death [12]. In addition, calcium hydroxide [Ca(OH)_2_] is used as an intracanal dressing for different indications, mainly as it has antimicrobial and antiendotoxin properties [13,14]. Its high alkalinity denatures proteins and damages microbial cell walls and cytoplasmic membranes [15].

Diverse herbal medicines are being studied to be used as endodontic irrigants or intracanal dressing [16]. Similarly, propolis is a resinous substance enriched by biologically active molecules; it is produced by bees using the sap of trees, and it has been studied for its antimicrobial, antioxidant, anti-inflammatory, and immunomodulatory properties. It contains a diverse range of biologically active compounds, such as flavonoids, phenolic acids, terpenes, and various other organic compounds [17], and the synergistic activity of these compounds justify its mechanism of action [18]. In endodontics, it was used as an irrigant and intracanal dressing [19] because of its efficacy against diverse dental microorganisms [17]. As far as we know, no studies have been performed on propolis’ effect on LTA capacity in macrophages’ proinflammatory cytokines production.

Consequently, the present study was performed to compare the effects of NaOCl, CHX, and the glycolic extract of propolis (GEP) as endodontic irrigants associated with Ca(OH)_2_, CHX, or Ca(OH)_2_ + CHX as intracanal dressings on the cytotoxic capacity of the LTA of *E. faecalis* to produce different macrophages’ proinflammatory cytokines. The null hypothesis was that NaOCl, CHX, and GEP associated with Ca(OH)_2_, CHX, or Ca(OH)_2_ + CHX have no effect on LTA capacity in macrophages’ proinflammatory cytokines production.

## 2. Materials and Methods

### 2.1. Preparation of Specimens

To perform this study, the project was presented to the research ethics committee for studies involving human beings of São Paulo State University to obtain authorization, which was approved under the protocol number (n 149.301). To collect extracted teeth by donors, an informed consent form was presented and signed by each donor. All the donated teeth were extracted for orthodontic and periodontal indications. A total of 108 freshly extracted human single-rooted teeth were used in this study. The teeth were selected based on dimensional and morphological similarities. Any teeth with caries, calcification, restoration or obturation were excluded.

The crown of each tooth was crosscut to standardize a length of 16 ± 0.5 mm for all specimens. Then, all specimens were instrumented using R25.08 (RECIPROC, VDW, Germany) at working length, which was determined by visualizing the instrument beyond the apical foramen. At that moment, 1% sodium hypochlorite was used to irrigate the canals by 5 mL for each canal third; then, all canals were filled with 17% ethylenediaminetetraacetic acid (EDTA) (Porto Alegre, RS, Brazil) for 3 min, activating with #30 K-file (RECIPROC, VDW, Germany) to remove the smear layer, and then washed out wth 10 mL of apyrogenic saline solution and dried with sterile paper points 25.08 (RECIPROC, VDW, Germany).

The sealing of the apical region of each specimen was accomplished by employing light-cured composite resin (Admira Fusion, Voco, Cuxhaven, Germany). Following this, a dual coating of nail polish was applied to the outer surface of the root, except the region of the cervical opening. Specimens were kept for 24 h at 37 °C until the nail polish dried; then, they were randomly distributed and fixed with chemically activated acrylic resin (JET, Artigos Odontológicos Clássico, Campo Limpo Paulista, SP, Brazil) in nine distinct 24-well microplates (TPP, Zollstrasse, Switzerland), in which n = 12 for each group.

**Figure 1:**
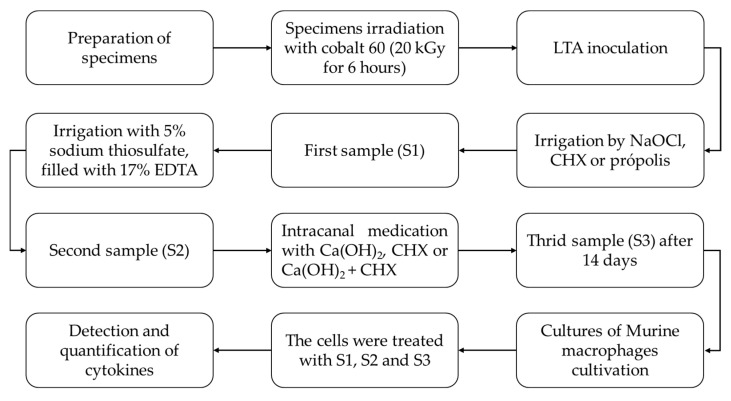
Flowchart of the study.

Figure 1 summarizes the flowchart of the study.

### 2.2. LTA Inoculation

After the specimens’ preparation, all materials and instruments used in this study were sent to be irradiated by gamma irradiation with cobalt 60 (20 kGy for 6 h) to neutralize pre-existing endotoxins. The LTA of *E. faecalis* (Sigma-Aldrich, St. Louis, MO, USA) was standardized in double-distilled apyrogenic water at a concentration of 250 µg/mL. Then, a total of three aliquots of 10 µL of the standardized LTA solution were inoculated in the root canal of each specimen. The specimens were kept at 37 °C with relative humidity for 24 h, and this inoculation was repeated after 24 and 48 h, totaling 72 h of incubation. After 24 h of the last inoculation, the specimens were instrumented using R40.06 and R50.05 (RECIPROC, VDW, Germany) and irrigated with one of the following irrigants (n = 36):

I. NaOCl group: 5 mL of 1% NaOCl for each third of the root canal was irrigated by a typical syringe and needle 30 G, totaling 15 mL for each specimen.
II. CHX group: the specimens were filled with 2% CHX; then, 5 mL of apyrogenic saline solution for each third of the root canal was irrigated via a typical syringe and needle (30 G), totaling 15 mL for each specimen.
III. GEP group: 5 mL of 12% glycolic extract of propolis for each third of the root canal was irrigated via typical syringe and needle (30 G), totaling 15 mL for each specimen.

The irrigation–aspiration was carried out using the NaviTip system (Ultradent Products Inc., South Jordan, UT, USA), and White Mac Tips (Ultradent) were attached to the cannula of the vacuum pump for aspiration.

Immediately after the instrumentation, the first sample (S1) was collected. The samples were collected by flooding the root canal with an apyrogenic saline solution, and a sample of 100 µL was collected using an insulin syringe and needle. Then, all specimens were irrigated with 5% sodium thiosulfate, filled with 17% EDTA for 3 min activating with #50 K-file, and then washed out with 10 mL of apyrogenic saline solution, in which the second sample was collected (S2), and all canals were dried with sterile paper points 50.05 (RECIPROC, VDW, Munich, Germany).

Lastly, the specimens were subdivided into nine groups, as detailed in Table 1, and treated by one of the following intracanal dressings (n = 12):

I. Ca(OH)_2_ group: The powder of Ca(OH)_2_ (Biodinamica Química e Farmacêutica LTDA, Ibiporã, Paraná, Brazil) was spatulated with apyrogenic saline solution with a proportion of 1:1 (100 mg of Ca (OH)_2_ powder with 100 µL of apyrogenic saline solution) over a sterilized glass plate until obtaining a smooth paste with no lumps (toothpaste-like consistency). Then, the paste was inserted into the root canal of the specimens. The paste consistency was toothpaste-like.
II. CHX group: 2% CHX gel (Biofórmula Manipulação, São José dos Campos, SP, Brazil), inserted into the root canal of the specimens, was used.
III. Ca(OH)_2_ + CHX group: The powder of Ca(OH)_2_ was spatulated with 2% CHX gel with a proportion of 1:1 (100 mg of Ca (OH)_2_ powder with 100 µL of 2% CHX gel) over a sterilized glass plate until a smooth paste with no lumps (toothpaste-like consistency) was obtained. Then, the paste was inserted into the root canal of the specimens.

All intracanal dressings were inserted with a lentulo spiral. The specimens were kept at 37 °C and relative humidity of 100%. After 14 days, the dressings were removed using 10 mL of apyrogenic saline solution, in which the third sample (S3) was collected at that moment.

### 2.3. Cells Cultivation and Cytokines Quantification

Cultures of murine macrophages (RAW 264.7) (Rio de Janeiro Cell Bank-APABCAM, Rio de Janeiro, RJ, Brazil) were cultivated in Dulbecco’s modified eagle medium (DMEM) (LGC Biotechnology, Cotia, Brazil) supplemented with 10% fetal bovine serum (FBS) (Invitrogen, New York, NY, USA). The incubation took place at 37 °C with 5% CO_2_ under standard atmospheric humidity conditions using cell culture flasks (TPP, Zollstrasse, Switzerland). Regular replacement of the culture medium occurred every 48 h until a state of subconfluence of the cells was reached, prompting transfer to a new cell flask. Subsequently, the cells underwent centrifugation at 2000 rpm for 5 min in a falcon-type tube.

Trypan blue (0.4%, Sigma-Aldrich, St. Louis, MO, USA) exclusion test was performed to quantify the number of viable cells. The cells were distributed 5 × 10^5^ and cultivated in 24-well microplates; then, 200 μL of DMEM medium was added and supplemented with 10% FBS containing 5 × 10^5^ viable cells. These plates were incubated (37 °C, 5% CO_2_) for 24 h for cell adhesion. Then, the supernatant was discarded, and the cells were washed with PBS.

The cells were treated with 30 µL of each sample (S1, S2 and S3) collected from root canals and incubated (37 °C, 5% CO_2_) for 24 h. The apyrogenic saline solution was used as the control group. After 24 h, supernatants were removed and frozen (−20 °C) for subsequent detection and quantification of cytokines (TNF-α, IL-6, IP-10, MIP-1α, G-CSF, and IL-1β) via immunosorbent assay (ELISA) [20].

Anti-TNF-α, anti-IL-6, anti-IP-10, anti-MIP-1α, anti-G-CSF, and anti-IL-1β (R&D Systems, NS) DuoSet kits were employed for conducting the ELISA assay according to the manufacturer’s recommendations. After obtaining the optical densities, the levels of cytokines (pg/mL) present in the culture supernatants of macrophages were determined using GraphPad Prism 5.0.

### 2.4. Statistical Analysis

All the data were submitted to a normality test and then analyzed with the one-way ANOVA test, complemented by the Tukey test, considering a significance level set at α  ≤  0.05, using GraphPad Prism 6 (La Jolla, CA, USA).

## 3. Results

### 3.1. S1 and S2

**Figure 2:**
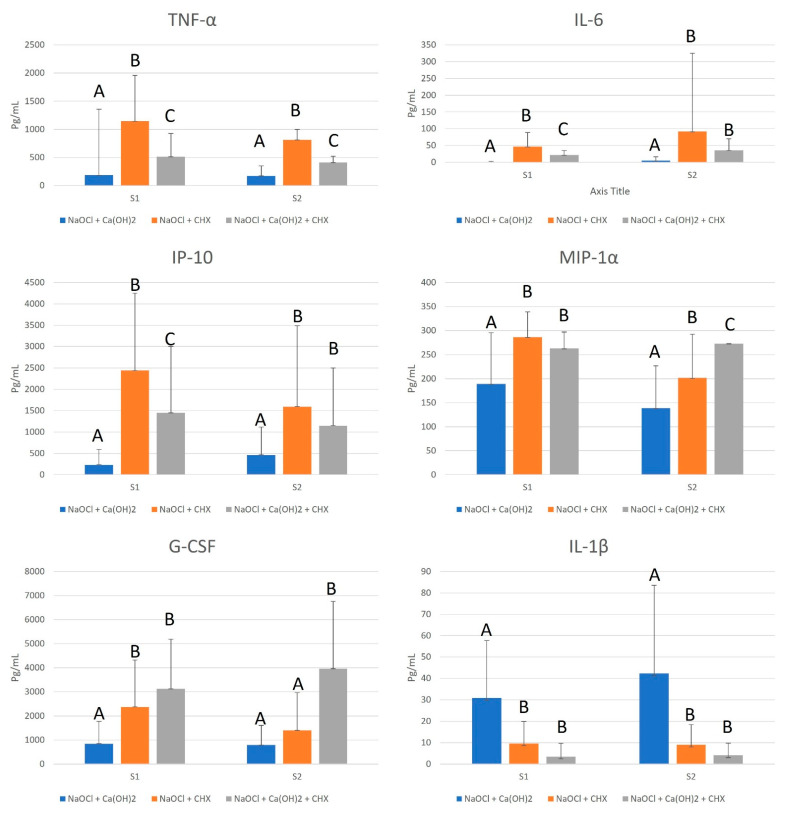
Mean values, standard deviation, and formation of homogeneous groups for the production of cytokines TNF-α, IL-6, IP-10, MIP-1α, G-CSF, and IL-1β (pg/mL) at S1 and S2. Different uppercase letters (A, B, and C) indicate a statistically significant difference.

The NaOCl group was the most effective in reducing the capacity of LTA in TNF-α, IL-6, IP-10, MIP-1α, G-CSF, and IL-1β production in S1 and S2, presenting a statistically significant difference to the other tested groups (*p* ≤ 0.05), as found in Figure 2.

GEP at 12% was more effective than 2% CHX in reducing the capacity of LTA in TNF-α, IL-6 and IP-10 production (*p* ≤ 0.05), and as effective as 2% CHX in reducing the capacity of LTA in MIP-1α, G-CSF, and IL-1β production in S1 (*p* > 0.05). However, in S2, 2% CHX was as effective as 1% NaOCl in reducing the capacity of LTA in G-CSF production (*p* > 0.05), and as effective as 12% GEP in reducing the capacity of LTA in IL-6, IP-10, and IL-1β production (*p* > 0.05), as shown in Figure 2.

### 3.2. S3

**Figure 3:**
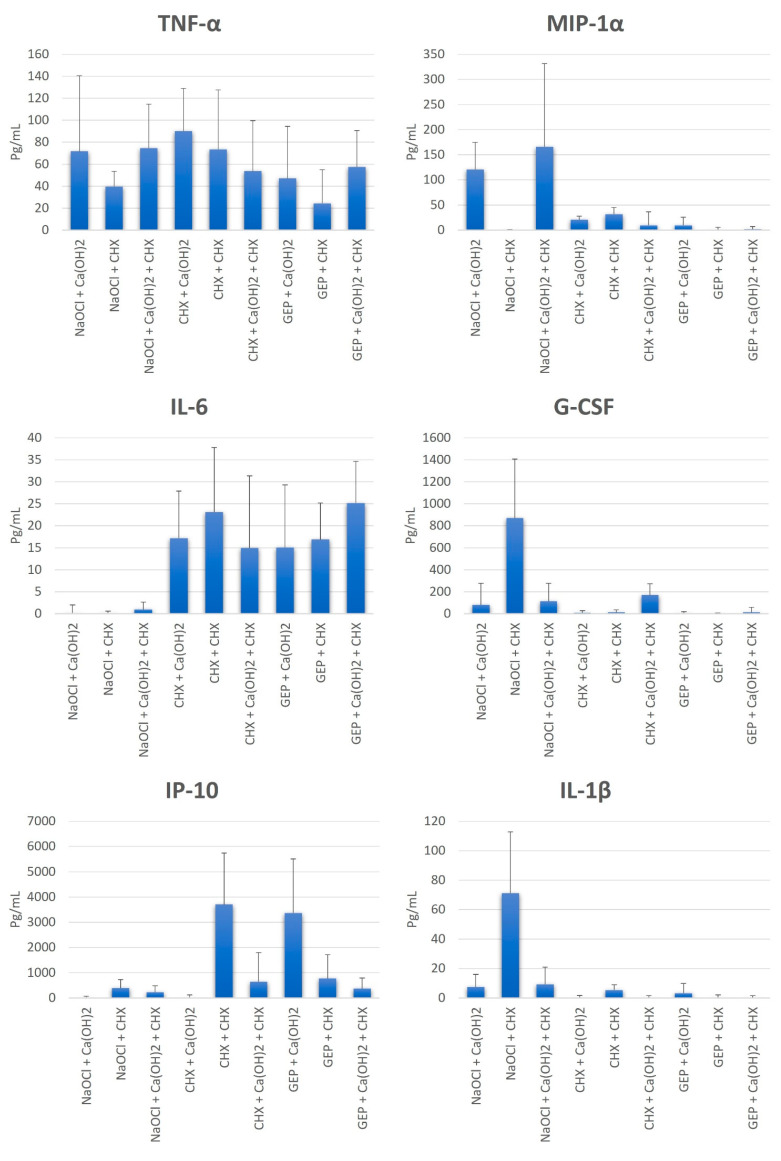
Mean values, standard deviation, and formation of homogeneous groups for the production of cytokines TNF-α, IL-6, IP-10, MIP-1α, G-CSF, and IL-1β (pg/mL) at S3.

All groups presented a mean production of TNF-α lower than the apyrogenic saline solution (control group) (165.6 pg/mL), with a statistically significant difference (*p* = 0.0005). The use of NaOCl with all the tested intracanal dressing was effective in reducing the capacity of LTA in IL-6 production, with a statistically significant difference (*p* < 0.0001) to the control group (Figure 3).

All groups were effective in reducing the capacity of LTA in IP-10 production, with a statistically significant difference (*p* < 0.0001) to the control group, except for the CHX + CHX and GEP + Ca(OH)_2_ groups. Moreover, all groups affected the LTA by presenting a mean production of MIP-1α, G-CSF, and IL-1β lower than the control group, with a statistically significant difference (*p* < 0.05), except for the NaOCl + Ca(OH)_2_ and NaOCl + Ca(OH)_2_ + CHX groups in MIP-1α, and the NaOCl + CHX group in G-CSF and IL-1β (Figure 3).

## 4. Discussion

The antiendotoxin of antimicrobial agents is of great relevance to the success of root canal treatment. The LTA of *E. faecalis* has been studied little in endodontics [20]. Therefore, this study was performed to evaluate the effect of three endodontic irrigants, including NaOCl, CHX, and GEP, associated with three intracanal dressings, including Ca(OH)_2_, CHX, or Ca(OH)_2_ + CHX, on the cytotoxic capacity of LTA in macrophages’ proinflammatory cytokines production.

In the present study, these macrophages’ proinflammatory cytokines were selected. As TNF-α is a characteristic protein of the acute phase of the inflammatory process, it initiates the production of other cytokines, increases vascular permeability, and recruits macrophages and neutrophils [21]. Interleukin 1β (IL-1β) is a potent pro-inflammatory cytokine, responsible for host defense against infections and capable of inducing tissue destruction reactions in the pulp and periapex [22]. Interleukin 6 (IL-6) is a multifunctional cytokine, important in both acute and chronic inflammation [23]. IP-10 and MIP-1α are chemokines that act at the site of infection, playing an important role in the recruitment and activation of leukocytes [24], and the granulocyte colony-stimulating factor (G-CSF) plays an important role in the proliferation and differentiation of neutrophil progenitor cells. Thus, these cytokines were selected in the present study due to their importance in the inflammatory and cytotoxic response.

In this study, reciprocating files were used to perform the instrumentation; this is in accord with a previous study [20]. The use of reciprocating or rotary instruments in this study is the same as that according to [25]. Reciproc and WaveOne reciprocating systems, in addition to the ProTaper system, are effective in removing the LPS and bacteria present in secondary/persistent endodontic infections. It was found that 1% NaOCl was the most effective endodontic irrigant in reducing the capacity of LTA in the production of all the tested pro-inflammatory cytokines in S1 and S2, presenting a statistically significant difference compared to the other tested groups (*p* ≤ 0.05). The efficacy of NaOCl may be explained by the fact that it deacylated the glycolipid moiety of the LTA of *E. faecalis*, which fails to activate TLR2, leading to the reduced production of inflammatory cytokines [26].

In addition, 12% GEP was more effective than 2% CHX in reducing the capacity of LTA in TNF-α, IL-6, and IP-10 production (*p* ≤ 0.05), and as effective as 2% CHX in reducing the capacity of LTA in MIP-1α, G-CSF, and IL-1β production in S1 (*p* > 0.05). This study is the first in the literature to evaluate the GEP effect of cytokines production, making the comparison of the present results with previous studies impossible. However, Neiva et al. (2014) [27] verified that the alcoholic extract of propolis was effective in reducing the LPS capacity in pro-inflammatory cytokines IL-1α, IL-6, IL-12, IL-15, G-CSF, TNF-α, MIP -1α, MCP-1, and IP-10 production by macrophages, osteoclasts, and odontoblasts, concluding that propolis extract suppresses the inflammatory response induced by LPS in key cells in the root canal system. Moreover, Wang et al. (2014) [28] analyzed the effects of Chinese propolis extract and found that its administration promoted a significant protective effect in attenuating histopathological changes and suppressing the secretion of inflammatory cytokines stimulated by LPS, such as IL-6 and TNF-α, in mice with endotoxemia, revealing potent anti-inflammatory activity of Chinese propolis.

After S2, the cytokine productions were maintained, in which 1% NaOCl had the lowest production, followed by 12% GEP and, finally, 2% CHX. Thus, it can be observed that the use of EDTA did not alter the irrigants’ effect in reducing the cytotoxic potential of LTA.

The second objective of the present study was to evaluate the effect of the combination of the previous endodontic irrigants with different intracanal dressings. The use of Ca(OH)_2_ as an intracanal dressing with all the tested irrigants presented good results in reducing the cytotoxic potential of LTA in cytokines production. This was confirmed in the literature, as Ca(OH)_2_ has an increased effect in terms of its reducing endotoxins and thus modulating the activation of proinflammatory cells such as macrophages [29]. Also, CHX was effective as an intracanal dressing in reducing the cytotoxic potential of LTA in cytokines production, except for in its combination with NaOCl as an irrigant on G-CSF and IL-1β. This may be attributed to the limited effectiveness of CHX against endotoxins [30].

The combination of Ca(OH)_2_ + CHX as an intracanal dressing presented the best results in this study with all the tested irrigants, except for in its use with NaOCl as an irrigant, in reducing the cytotoxic potential of LTA in MIP-1α production. However, this combined dressing has been tested previously, and it was proven effective in reducing macrophages’ proinflammatory cytokines production, including TNF-α, IL-1β, IL-6, and G-CSF [20], as it improved the removal of bacteria and endotoxins from infected root canals [31,32].

One point to be emphasized here is the impact of the use of intracanal medication on LTA levels. In multiple-visits treatment, it is noted that the LTA level is reduced significantly in comparison with single-visit treatment of secondary/persistent endodontic infection [13], thereby reducing the LTA-induced production of cytokines [20].

Lastly, the null hypothesis of this study was rejected as NaOCl, CHX, and GEP associated with Ca(OH)_2_, CHX, or both of them were effective in reducing the cytotoxic potential of LTA in macrophages; pro-inflammatory cytokines production. This study suggests the use of NaOCl as an irrigant in combination with Ca(OH)_2_ + CHX as the best protocol still, it indicates the use of GEP as an alternative irrigant with the same intracanal dressing as GEP is a natural product with good antimicrobial action and biocompatibility [17], and it presented promising results in the present study.

It is worth noting that NaOCl and CHX are widely used in endodontics as irrigants, and the literature has plenty of studies evaluating both irrigants. However, the use of propolis in endodontics is still largely experimental and primarily tested in in vitro settings [19,33]. This limitation should be considered when considering the aims and findings of this study. The promising in vitro results of GEP suggest that further research, including clinical trials, is needed to understand its potential and efficacy fully in endodontic applications. These findings support the integration of natural products like propolis in endodontic treatments, offering potential benefits in managing endodontic infections and inflammation. However, the need for further clinical research to validate these in vitro findings cannot be overstated.

## 5. Conclusions

- Sodium hypochlorite associated with calcium hydroxide + chlorhexidine was effective in reducing the LTA-induced production of TNF-α, IL-6, IP-10, G-CSF, and IL-1β, indicating its superior anti-inflammatory action.
- Propolis associated with calcium hydroxide + chlorhexidine was effective in the reducing LTA-induced production of TNF-α, IL-6, IP-10, MIP-1α, G-CSF, and IL-1β, highlighting its potential as a natural alternative endodontic irrigant.
- Chlorhexidine has limited efficacy in reducing the LTA-induced production of macrophage’s pro-inflammatory cytokines.

## Figures and Tables

**Table 1:** The experimental groups description.

Experimental Groups	Endodontic Irrigant	Intracanal Dressing
NaOCl + Ca(OH)_2_	1% NaOCl	Ca(OH)_2_
NaOCl + CHX	1% NaOCl	CHX
NaOCl + Ca(OH)_2_ + CHX	1% NaOCl	Ca(OH)_2_ + CHX
CHX + Ca(OH)_2_	2% CHX	Ca(OH)_2_
CHX + CHX	2% CHX	CHX
CHX + Ca(OH)_2_ + CHX	2% CHX	Ca(OH)_2_ + CHX
GEP + Ca(OH)_2_	12% GEP	Ca(OH)_2_
GEP + CHX	12% GEP	CHX
GEP + Ca(OH)_2_ + CHX	12% GEP	Ca(OH)_2_ + CHX

Abbreviations: NaOCl: sodium hypochlorite; Ca(OH)_2_: calcium hydroxide; CHX: chlorhexidine.

## Data Availability

Data are available upon request (d.d.s.amjad@gmail.com).
